# Pregnancy outcomes in women with idiopathic thrombocytopenic purpura

**Published:** 2012-09

**Authors:** Fakhrolmolouk Yassaee, Roghieh Eskandari, Zohreh Amiri

**Affiliations:** 1 *Department of Obstetrics and Gynecology, Genomic Research Center, Shahid Beheshti University of Medical Sciences, Taleghani Hospital, Tehran, Iran.*; 2 *Department of Obstetrics and Gynecology, Shahid Beheshti University of Medical Sciences, Taleghani Hospital, Tehran, Iran.*; 3 *Department of Basic Sciences, Faculty of Nutrition and Food Technology, Shahid Beheshti University of Medical Sciences, Tehran, Iran.*

**Keywords:** *Pregnancy outcomes*, *Idiopathic thrombocytopenic purpura*, *Neonatal outcome*

## Abstract

**Background:** Idiopathic thrombocytopenic purpura (ITP) is a disease that commonly affects women of reproductive age and is associated with maternal and fetal complications.

**Objective: **The aim of the present study was to report the perinatal outcome in pregnant women with ITP.

**Materials and Methods:** Twenty one pregnant women with ITP admitted in a teaching hospital in Tehran, from October 2008 to February 2010, were enrolled in this prospective historical cohort study; course and perinatal outcome of pregnancies were studied.

**Results:** Seven (33.3%) cases had been diagnosed before pregnancy, while the other fourteen (66.7%) were diagnosed during pregnancy. During hospitalization, thirteen (62%) patients required treatment, eight (61.5%) of them with steroids, two (15.3%) received intravenous immunoglobulin (IVIG), and three (23%) were treated with steroids and IVIG. Three babies were delivered vaginally (14.3%), seventeen (81%) through cesarean section and one patient aborted her fetus. Nine mothers (42.9%) had platelet counts <50000/ml at the time of delivery; but postpartum hemorrhage occurred in 4 (19%) women and one women received platelet transfusion during cesarean section. Six (28.6%) women developed gestational diabetes. Pregnancy was complicated by preeclampsia in one woman and by abruptio placenta in another. One pregnancy terminated in intrauterine fetal death. Seventeen infants (89.5%) had normal platelet counts, and two (10.5%) had moderate thrombocytopenia. No infant showed signs of hemorrhage, but 2 neonates (10.5%) were diagnosed with intrauterine growth restriction.

**Conclusion:** Rate of gestational diabetes in pregnant women with ITP is higher than the general population. Rate of gestinational diabetes is 3-5% and postpartum hemorrhage is 5-7% in general. Postpartum hemorrhage is common in these women. Severe thrombocytopenia and bleeding in the newborns are uncommon.

## Introduction

Idiopathic thrombocytopenic purpura, (ITP) usually results from a cluster of IgG anti bodies directed against one or more platelet glycoproteins. ITP is a chronic disease in young women and rarely resolves spontaneously. Its incidence is 1/1000-10.000 pregnancies, (1). It is certainly not unusual for women who have been in clinical remission for several years to have recurrent thrombocytopenia during pregnancy. Although there is no evidence that ITP is aggravated during pregnancy but this disorder can cause increased bleeding intrapartum or post-partum, (1-3). 

Also, platelet associated IgG antibodies cross the placenta and may cause thrombocytopenia in the fetus and neonate. Maternal platelet count cannot predict neonatal platelet count. So neonatal platelet count can be predicted through fetal scalp blood sampling during labor or by percutaneous umbilical blood sampling (PUBS).These two procedures are invasive and may be complicated by fetal scalp hematoma or fetal bradycardia and are not considered necessary (1, 4). 

There are many controversies about the type of delivery in these women, because of the lack of evidence to support the role of cesarean section in the prevention of neonatal intracranial hemorrhage (5), although there is report of benefit of cesarean section in these women (4, 5). In this study we have documented the course of disease and the outcome of pregnancy in pregnant women with ITP.

## Materials and methods

Twenty one pregnant women with ITP admitted in a teaching hospital in the North of Tehran, from October 2008 to February 2010 were enrolled in this prospective historical cohort study. The study was approved by Taleghani Hospital ethics committee. Some of these women were diagnosed by hematologist as ITP before pregnancy and were under the treatment and after becoming pregnant they were under our observation in the obstetric ward and the others were diagnosed as thrombocytopenia in pregnancy and after consultation by hematologist and exclusion of gestational thrombocytopenia, systemic lupus erythematosis, lymphomas, leukemias HIV, positive patients, hypertensive disorder of pregnancy and diagnosis of ITP were admitted in our ward.

During the course of hospitalization all clinical manifestations, results of relevant laboratory tests, treatment protocols and response to therapy were carefully documented by members of the study team comprising of an obstetrician, a hematologist, and a neonatologist. Emergent pre- and postpartum complications including, bleeding during pregnancy or post-partum hemorrhage, gestational diabetes, preeclampsia, intrauterine growth restriction or intrauterine fetal death, preterm labor, type of delivery, neonatal condition and development of neonatal thrombocytopenia were recorded. 

**Statistical analysis**

Data were collected and analyzed by SPSS software, version 16.

## Results

The mean (±SD) age of women was 28.9±7.17 (range 18-45) years. The mean parity was 0.92±1.65 (range 0-6). The level of education in these women was from primary school up to high school. Out of 21 patients with idiopathic thrombocytopenic purpura, seven (33.3%) had already been diagnosed with ITP before getting pregnant. They were under the care of hematologist and were already on treatment with prednisolone. The other fourteen (66.7%) were diagnosed during pregnancy; one of them was diagnosed in the first trimester, 7 (33.3%) in the second trimester and 6 (28.6%) in the third trimester. 

During hospitalization, thirteen patients (62%) had very low platelet counts (<50.000/ml) and required treatment but in 8 patients (38%) the thrombocytopenia was mild with platelet counts consistently >50,000/ml needing no therapy. Out of these 13 patients, eight (61.5%) required steroids, two (15.3%) received IVIG and 3 (23%) were given both IVIG and steroids. 

During the antenatal period, one patient aborted her fetus, six (28.6%) mothers developed gestational diabetes, from which 5 of them were under treatment with corticosteroid. In one patient the pregnancy was complicated by preeclampsia, in one by abruptio placenta and in another one by intrauterine fetal death (IUFD). 

At the time of delivery 12 (57.1%) patients had moderate thrombocytopenia (>50,000/ml) and 9 patients (42.9%), had severe thrombocytopenia (<50,000/ml) (Table I), and 4 (19%) developed post-partum hemorrhage; however none required a blood transfusion. One patient with platelet count 48000/ml developed excessive bleeding needed platelet transfusion during cesarean section. 

There were no premature deliveries (<37 weeks of gestation). Of 21 pregnant women, 3 (14.3%) delivered vaginally, 17 (81%) underwent cesarean section, all for obstetric reasons (Table II). Two babies, (10.5%) developed intrauterine growth restriction (IUGR). Two infants (10.5%) had thrombocytopenia (<100,000/ml) and 17 (89.5%) had normal platelet count (Table III). No infant showed sign of hemorrhage.

**Table I:** Platelet count of women with ITP at the time of delivery

**Platelet count**	**n (%)**
<50,000/ml	9 (42.9)
>50,000/ml	12 (57.1)
Total	21 (100)

**Table II:** Route of delivery in 20 women with ITP

**Route of delivery **	**n (%)**
Vaginal	3 (15)
Cesarean section	17 (85)
Total	20 (100)

Abortion is not considered.

**Table III:** Infant platelet count

**Platelet count**	**No (%)**
>100,000/ml	17 (89.5)
<100,000/ml	2 (10.5)
Total*	19 (100)

* One patient aborted.

* One patient developed IUFD.

## Discussion

Findings of our study reveal that, pregnancy in women with idiopathic thrombocytopenic purpura can be safe. Several other researchers have reported similar results in previous studies, (2-5).

The first line of treatment in ITP is corticosteroid therapy (1, 5-7, 12), however patients on steroids have an increased risk of developing gestational diabetes mellitus. In our study out of the six women who developed gestational diabetes mellitus, five had been treated with steroids; this is consistent with the study by Suri *et al* (15).

Maternal platelet antibody can cross the placenta and induce neonatal thrombocytopenia; in this study 2 babies, (10.5%) developed moderate thrombocytopenia, (platelet count <100,000/ml) however, as seen in previous studies, there was no serious hemorrhagic complication, such as intracranial hemorrhage and none of them required treatment (2-4, 11, 13). Maternal platelet count cannot predict neonatal platelet count, which can be obtained through fetal scalp sampling during labor or by percutaneous umbilical blood sampling (PUBS) prior to delivery. 

These two procedures are invasive, fraught with complications such as fetal scalp hematoma or fetal bradycardia, and not considered necessary (1). If neonatal thrombocytopenia does occur it is diagnosed and managed postpartum. Consequently, none of the women in our study were subjected to PUBS or fetal scalp blood sampling. Because of the risk of maternal and/or fetal hemorrhage, choosing the ideal route of delivery in women with ITP has been a matter of debate during the previous decades. 

Traditionally, most of these mothers were delivered by cesarean section however there are no data to support the superiority of cesarean section in lowering the risks for the thrombocytopenic fetus as compared to vaginal delivery, (4-6, 8, 10, 12, 13, 17). In our study, 17 of 20 deliveries (81%) were cesarean sections; similar to previous reports, all of them were performed for obstetric indications, and none because of thrombocytopenia, (2, 12, 14). While doing a cesarean section on one of our patients with a platelet count of 48000/ml, we encountered profuse bleeding, which necessitated a platelet transfusion. Although 9 patients, (42.9%) had severe thrombocytopenia none of them required a blood transfusion; however 4, (19%), developed postpartum hemorrhage. During antenatal period one patient developed preeclampsia. 

This complication has been reported in previous studies as well, (3, 15). Intrauterine fetal death has been reported in some studies we also lost one fetus (3, 5). Abruptio placenta occurred in one of our patients; this complication is reported in the study by Ali *et al* (3). We did not have any cases of preterm delivery in contrast to a study which is done by Belkin and colleagues in 2009 who reported a high rate of preterm delivery (13). 

## Conclusion

Our findings indicate that the rate of gestational diabetes is higher than the general population in pregnant women with thrombocytopenia who are treated with steroids. However our results do not show an increase in the rate of preeclampsia, preterm labor and/or adverse effects on the neonate. Cesarean section should be performed for obstetric indications only. We conclude that a safe outcome of pregnancy in women with ITP requires teamwork between hematologists, obstetricians and neonatologists. Our limitation was small sample size, because this study was done in 2 years and was prospective, but previous studies were done retrospectively
